# USE OF SPATIOTEMPORAL GAIT PARAMETERS TO DETERMINE RETURN TO SPORTS AFTER ACL RECONSTRUCTION

**DOI:** 10.1590/1413-785220162402147450

**Published:** 2016

**Authors:** GUSTAVO LEPORACE, LEONARDO METSAVAHT, GABRIEL ZEITOUNE, THIAGO MARINHO, TAINÁ OLIVEIRA, GLAUBER RIBEIRO PEREIRA, LISZT PALMEIRA DE OLIVEIRA, LUIZ ALBERTO BATISTA

**Affiliations:** 1. Universidade Federal do Rio de Janeiro, Rio de Janeiro, RJ, Brazil; 2. Instituto Brasil de Tecnologia da Saúde, Rio de Janeiro, RJ, Brazil; 3. Universidade do Estado do Rio de Janeiro, Rio de Janeiro, RJ, Brazil

**Keywords:** Wounds and injuries, Knee, Sports

## Abstract

**Objective:**

: To compare gait spatiotemporal parameters of healthy and ACL reconstructed subjects in order to classify the status of gait normality.

**Methods:**

: Fourteen healthy subjects and eight patients submitted to ACL reconstruction walked along a walkway while the lower limbs movement was captured by an infrared camera system. The frames where the initial contact and toe-off took place were determined and the following dependent variables, which were compared between groups through the Mann-Whitney test (a=0.05) were calculated: percentage of time in initial double stance, percentage of time in single stance, percentage of time in terminal double stance, stride length and gait velocity. Initially, all variables were compared between groups using a Mann-Whitney test. A logistic regression was applied, including all dependent variables, to create a model that could differentiate healthy and ACL reconstructed subjects.

**Results:**

: ACL reconstructed group showed no differences in any spatiotemporal parameter of gait (p > 0.05) in relation to the control group, although the angular kinematic differences of the knee remained altered, as evidenced in a study with a similar sample.

**Conclusion:**

: The regression classified all subjects as healthy, including the ACL reconstructed group, suggesting the spatiotemporal variables should not be used as the sole criterion of return to sports activities at the same level as prior to injury. Level of Evidence III, Case Control Study.

## INTRODUCTION

Anterior cruciate ligament (ACL) injury is among the most common injuries in sports traumatology^1^ and the ligament instability resulting from it has a significant association with secondary comorbidities and recurrent injuries. In the United States, approximately 90% of ACL injuries are treated by ligament reconstruction surgery. Although the middle and long term results are not as good as expected by the orthopedic community, still it is considered the best option. According to Lohmander et al.,^2^ 50% to 100% of the individuals with anterior cruciate ligament injury, regardless of ligament reconstruction, will present pain, functional limitations and radiographic signs of osteoarthritis (OA) in the injured knee within 12-20 years after the injury event. Paterno et al.,^3^ in a cohort study, reported that incidence of recurrent knee sprains in the first year after ACL injury is 15 times greater than the primary event. They also suggest that after ACL injury and reconstruction, the incidence of injuries in contralateral knee is higher than expected in uninjured recreational athletes.^3^

It seems that the high incidence of recurrent sprains after ACL reconstruction may be due to residual weaknesses not restored during the rehabilitation process.^4^ Biomechanics studies have shown that injured individuals tend to adapt the lower limb movement pattern for several tasks and present impairment in force generation even after two years reconstruction surgery.^5^ Nevertheless, in literature we could not find any consensual agreement about what criteria should be used to allow returning to sports activities. A recent systematic review by Barber-Westin and Noyes^4^ found that allowing return to sports activities should be based on clinic parameters, like ligament laxity on exam, the muscle recovery compared to contralateral limb and some specific functional tests. However, these criteria are subjective and also do not allow identification of potential risks for future comorbidities or re-injuries,^6^^,^ 7 so, approaches that could measure residual deficits would be preferable. 

Change in gait patterns have been identified even after ACL reconstruction (ACL-R) and rehabilitation.^8^^,^ 9 It has been shown that ACL-R subjects tend to present different knee angles during gait even one year after surgery. The main altered variables were related to the increase of adduction and rotation of the knee, with no alterations in the sagittal plane.^9^ Butler et al.^10^ reported that altered knee frontal and transverse planes angles during gait are related to premature development of knee OA.^11^^-^ 13

For clinical practice the evaluation of complex movements is limited by the high cost of equipment, but spatiotemporal gait parameters are pointed as objective clinical predictors of functionality and at low cost technology.^14^^,^ 15 However, it is not known whether these parameters could be used as an initial screening to the normalization of the gait after rehabilitation. Therefore, the aim of this study was to compare the spatiotemporal parameters of healthy and ACL-R subjects and classify the status of normality.

## MATERIALS AND METHODS

Twenty-two subjects, 14 in the control group (CG) and eight in the anterior cruciate ligament reconstruction group (ACL-R), with similar anthropometric characteristics participated in this study. (Table 1) The mean time from ACL-R group surgery was 11.2 ± 2.4 months (ranging between 9 and 15 months). All surgeries were performed by the same surgeon (L.M) using the same technique, with knee flexor tendons double-band autograft, by transtibial approach. The graft was proximally fixed with bioabsorbable transfemoral pins and with bioabsorbable interference screws in the tibia (Arthrex, USA). Existing meniscus or cartilage injuries were corrected with adequate techniques for each situation. No acute injuries were operated, so all patients presented normal knee range of motion and no inflammatory process signs before the surgery. The main complaint was knee instability with giving way episodes on daily living activities. All patients underwent similar rehabilitation programs, starting passive and active mobilization one day after surgery. Partial load with crutches was allowed within 5 days and total discharge after 10 to 21 days, as tolerated. 

**Table 1.** Anthropometric data of the sample and p value of comparison between them. Values reported as mean ± standard deviation.

**Parameters**	**CG**	**ACL-R**	**P - value**
Age (years)	27.3 ± 2.7	33.1 ± 11.1	0.288
Body mass (kg)	82.1 ± 9.5	82.1 ± 7.4	0.365
Height (cm)	180.4 ± 4.4	182.3 ± 2.9	0.771

The inclusion criteria for the CG were scoring over 90% of the International Knee Documentation Committee (IKDC) Subjective Knee Form^16^ and Lower Extremity Functional Scale.^17^ Subjects with orthopedic and neurologic injuries history or lower limb pain were excluded from the CG. All participants signed an informed consent form allowing participation in the study. This study was approved by the State University of Rio de Janeiro Research Ethics Council (number: 053/2009).

Subjects walked seven times at a self-selected speed along an eight-meters walkway. The first three laps were not measured to allow familiarization with the task and instrumentation. The last four laps were evaluated to capture four gait cycles of lower limb kinematic data, using the right limb in the CG and the injured limb in the ACL-R group. 

**Figure 1.**
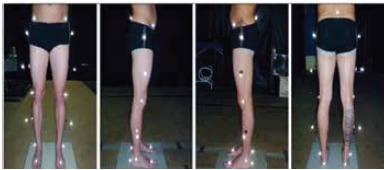
Marker set used in the data collection.

To allow the data collection 17 reflexive markers were positioned on sacrum, anterior-superior iliac spines, greater trochanters, femur lateral condyles, lateral malleolus, second metatarsal heads and calcaneal posterior region. Five cm wands were also set at the middle thighs and shanks.^18^ (Figure 1)

The markers were captured by a four cameras motion analysis system (MaxPro version 1.4.2.1, INNOVISION Systems, USA), with 60 Hz sample rate. The 2D coordinates of each marker were captured by the cameras and transformed into 3D global coordinates by Direct Linear Transformation algorithm.

**Figure 2.**
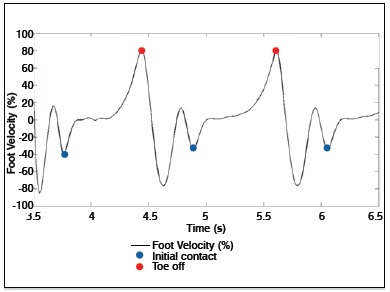
Toe-off and initial contact exemples detections by Foot Velocity Algorithm

The signs were filtered by a low pass 2^nd^ order Butterworth filter, applied in the forward and reverse directions to avoid phase distortions, with a cut-off frequency of 6 Hz. To determine the beginning and the final of each cycle, the Foot Velocity Algorithm was used.^19^ (Figure 2)

After identification of the initial contact and toe-off events for each gait cycle of each subjects, the following dependent variables were calculated: stance time percentage (% St), swing time percentage (% Sw), initial double limb stance time percentage (% IDS), single limb stance time percentage (% SS) terminal double limb stance time percentage (% TDS), stride length (SL) and gait velocity (Vel). All of data processing were performed in a tailor-made routine at MATLAB version 7.8.0 (The Mathworks, USA).

### Statistical analysis

The average of each dependent variable in the four gait cycles were calculated and compared between two groups using a Mann Whitney test, with the significance level set at 0.05. The effect size was calculated for each variable.^20^ Values above 0.8 were considered high and under 0.5 were considered low.^20^ The reliability of the spatiotemporal parameters was tested using an Intraclass Correlation Coefficient (ICC), type 2.1. The statistical analyses were performed in GraphPad Prism 5.00 software (GraphPad Software, San Diego, CA, USA).

To classify the individuals gait pattern, a logistic regression (LR) was used based on Schumacher et al.^21^



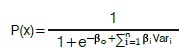



where b_o_ is the intercept, b_1_ are the coefficients associated with the explanatory variables (Var). The maximum likelihood technique was used to estimate the b coefficients.

The classificatory threshold was defined at 0.5. The stepwise approach was used to find the best model of LR, considering all possible predictor variables, through Akaike Information Criteria (AIC). The LR performance was evaluated by leave-one-out cross-validation technique.

## RESULTS

The reliability of the spatiotemporal parameters results are described in Table 2. The data reliability values ranged from moderate (between 0.5 and 0.6) to excellent (above 0.9). The highest reliability was obtained to stride length and gait velocity data and the lowest to % St (0.58).

**Table 2.** Gait spatiotemporal parameters among of four cycles for each group Intraclass Correlation Coefficient (ICC). The results are expressed as ICC(95% CI).

**Parameters**	**CG**	**ACL-R**
St	0.576 (0.044 - 0.848)	0.659 (0.012 - 0.923)
IDS	0.797 (0.543 - 0.927)	0.832 (0.501 - 0.962)
SS	0.819 (0.590 - 0.935)	0.780 (0.346 - 0.951)
TDS	0.768 (0.476 - 0.917)	0.693 (0.088 - 0.931)
SL	0.910 (0.797 - 0.968)	0.972 (0.916 - 0.994)
Vel	0.804 (0.558 - 0.930)	0.926 (0.780 - 0.983)

St: Stance; IDS: Initial Double Stance; SS: Single Stance; TDS: Terminal Double Stance; SL: Stride Length; Vel: Velocity.

No differences were found among variables between both groups. (Table 3) The effect size was low (below 0.4) for all variables. The logistic regression identified that all ACL-R group individuals were classified as healthy. No variable was described as significant in the final model to identify possible differences between groups.

**Table 3.** Comparison (mean ± standard deviation) between both groups for all dependent variables.

**Parameters**	**CG**	**ACL-R**	**p value**	**Effect size**
St (%)	60.0 ± 0.6	59.9 ± 1.2	0.657	0.12
IDS (%)	10.6 ± 1.0	10.4 ± 1.1	0.626	0.22
SS (%)	39.2 ± 1.1	39.4 ± 1.1	0.918	0.16
TDS(%)	10.2 ± 0.9	10.1 ± 1.2	0.923	0.05
SL (m)	1.32 ± 0.07	1.36 ± 0.09	0.473	0.37
Vel (m/s)	1.21 ± 0.08	1.22 ± 0.09	0.891	0.08

St: Stance; IDS: Initial Double Stance; SS: Single Stance; TDS: Terminal Double Stance; SL: Stride Lenght; Vel: Velocity.

## DISCUSSION

According to Kuo and Donelan,^22^ alterations in spatio temporal parameters can lead to an increased mechanical work and, consequently, greater energy expenditure to walk. For example, a decrease in the single limb stance time can lead to an increased opposite limb swing velocity, decreasing the impact generated at initial contact.^22^ However, this strategy increases energy expenditure.^23^ Therefore, the study of spatiotemporal parameters is necessary to evaluate the strategies to perform an efficient gait.

Studies suggest that spatiotemporal parameters, in individuals without neurologic injuries, are mainly influenced by biomechanical variables in the sagittal plane,^24^^,^ 25 whereas discrete changes in frontal and transverse planes can reflect in few changes in gait dynamics.^22^ Gait pattern studies have shown that within six months after ACL reconstruction, the kinematics in the sagittal plane of the reconstructed limb returns to the level of healthy subjects.^8^^,^ 9 However, changes in frontal and transverse plane seem to keep changed even at the end of physiotherapy rehabilitation.^8^^,^ 9 These change can be associated to an increased risk of premature degeneration of knee cartilage,^11^^,^ 12 and to the early onset signs of osteoarthritis.^2^ Thus, one can infer that it is possible that even after normalization of gait spatiotemporal variables it may still exist biomechanical changes in the lower limbs^5^ that can lead to new or secondary injuries.^3^

In the present study, the evaluated subjects were the same as of the Leporace et al.^9^ study, in which all of individuals one year after ACL reconstruction presented abnormal gait kinematics. In that investigation the main altered variables were related to lower limbs alignment, where they found an increase in knee varus and internal rotation, with a normalized flexion/extension pattern. In the present study, in which gait spatiotemporal variables were assessed, there were no differences compared to a control group (Table 2) and all subjects were classified as healthy by logistic regression. 

These findings are supported by the literature. Georgoulis et al.^26^ and Gao et al.^8^ did not find differences in gait velocity between ACL-reconstructed subjects between four to 15 months and a control group. Gao et al.^8^ and Knoll et al.^27^ also did not find differences for stride and step length one year after surgery. Minning et al.^28^ showed that spatiotemporal variables normalize about two to three months after ACL reconstruction. Despite gait spatiotemporal parameters normalization, the current evidences shown that three month after injury there are still residual deficits, which may remain even one year after surgery.^5^^,^ 29 ^,^ 30 Therefore, the use of these variables as criteria to return sports activities must be avoided, since as noted, the investigated parameters in current study was not sensitive enough to identify the ACL group that still presented important deficits in gait kinematics.

Despite the agreement with other studies, the small sample size used in this study does not allow the generalization of the results. To overcome this limitation, we used the effect size calculation to complement the statistical results. The lack of control of the physical therapy process is also a limitation, although the protocols used for rehabilitation were similar. 

## CONCLUSION

The ACL-R group did not show differences in spatiotemporal gait parameters related to a control group. The logistic regression classified all subjects as non-injured. This finding indicates that spatiotemporal variables are not good parameters to differentiate knee gait biomechanics of ACL-R subjects from healthy ones and should not be used as criteria to determine the return to sports after anterior cruciate ligament reconstruction.
